# Distinct childhood neurodevelopmental trajectories following very preterm birth

**DOI:** 10.1192/j.eurpsy.2023.846

**Published:** 2023-07-19

**Authors:** M. Solerdelcoll Arimany, C. Nosarti

**Affiliations:** 1 Department of Child and Adolescent Psychiatry and Psychology, Institute of Neuroscience, Hospital Clínic de Barcelona, Barcelona, Spain; 2 Department of Child and Adolescent Psychiatry, Institute of Psychiatry, Psychology & Neuroscience, King’s College London; 3 Department of Child and Adolescent Psychiatry, Institute of Psychiatry, Psychology and Neuroscience. King’s College London, London, United Kingdom

## Abstract

**Introduction:**

Very preterm birth (VPT; <32 weeks’ gestation) constitutes itself an environmental risk factor for a wide range of severe mental disorders. Particularly, 25% of VPT screen positively for autism spectrum disorder (ASD) and often present with co-occurring developmental difficulties, making it challenging to identify those likely to develop ASD traits. Therefore, neurodevelopmental trajectories associated with ASD outcomes need to be identified.

**Objectives:**

Here, we investigated infant-to-childhood ASD traits trajectories, and their association with neurodevelopmental comorbidities, in a sample of VPT children screening positively and negatively on the Modified Checklist for Autism in Toddlers (M-CHAT).

**Methods:**

VPT individuals from the Evaluation of Preterm Imaging study (ePrime) underwent behavioural assessments at 2 (M-CHAT and Bayley Scales of Infant Development; N=451) and 4-7 years (Social Responsiveness Scale (SRS-2); N=251). To furtherly assess the presence of comorbid neurodevelopmental disorders at children aged 4-7 years, further assessments of cognitive (WPPSI), ADHD (ADHD-RS-IV scale), and emotional and behavioural problems (SDQ and ECBQ) were conducted.

**Results:**

Findings of the ePRIME 4–7-year follow-up substudy will be presented. VPT children will be grouped using M-CHAT scores, as they reportedly show distinct neurodevelopmental characteristics. Preliminary results showed that ASD traits in infancy are associated with increased neurodevelopmental impairment.

**Conclusions:**

VPT infants may be an undescribed “at risk of ASD” or ASD cluster, with clinical features and comorbid neurodevelopmental disorders that differentiate them from other “at-risk” populations. Our findings support the need for routine ASD and ADHD assessments in VPT infants at preschool but also at school ages; and highlight the importance of interpreting ASD screenings in combination with other developmental measures when assessing VPT children. Our results could guide clinicians and researchers to offer personalised interventions aimed at supporting children’s development based on their distinct phenotypic presentations. Further research is needed to develop more accurate screening tools.

**Disclosure of Interest:**

None Declared

